# A Police and Insurance Joint Management System Based on High Precision BDS/GPS Positioning

**DOI:** 10.3390/s18010169

**Published:** 2018-01-10

**Authors:** Wenwei Zuo, Chi Guo, Jingnan Liu, Xuan Peng, Min Yang

**Affiliations:** 1 Global Navigation Satellite System Research Center, Wuhan University, Wuhan 430079, China; 2011301610151@whu.edu.cn (W.Z.); jnliu@whu.edu.cn (J.L.); 2 Traffic Management Research Institute of the Ministry of Public Security, Wuxi 213000, China; pengx_tmri@163.com; 3 School of Resource and Environmental Sciences, Wuhan University, Wuhan 430079, China; yangmin2003@whu.edu.cn

**Keywords:** lane level map, high precision positioning, traffic management, insurance survey, Internet of Vehicles (IoV)

## Abstract

Car ownership in China reached 194 million vehicles at the end of 2016. The traffic congestion index (TCI) exceeds 2.0 during rush hour in some cities. Inefficient processing for minor traffic accidents is considered to be one of the leading causes for road traffic jams. Meanwhile, the process after an accident is quite troublesome. The main reason is that it is almost always impossible to get the complete chain of evidence when the accident happens. Accordingly, a police and insurance joint management system is developed which is based on high precision BeiDou Navigation Satellite System (BDS)/Global Positioning System (GPS) positioning to process traffic accidents. First of all, an intelligent vehicle rearview mirror terminal is developed. The terminal applies a commonly used consumer electronic device with single frequency navigation. Based on the high precision BDS/GPS positioning algorithm, its accuracy can reach sub-meter level in the urban areas. More specifically, a kernel driver is built to realize the high precision positioning algorithm in an Android HAL layer. Thus the third-party application developers can call the general location Application Programming Interface (API) of the original standard Global Navigation Satellite System (GNSS) to get high precision positioning results. Therefore, the terminal can provide lane level positioning service for car users. Next, a remote traffic accident processing platform is built to provide big data analysis and management. According to the big data analysis of information collected by BDS high precision intelligent sense service, vehicle behaviors can be obtained. The platform can also automatically match and screen the data that uploads after an accident to achieve accurate reproduction of the scene. Thus, it helps traffic police and insurance personnel to complete remote responsibility identification and survey for the accident. Thirdly, a rapid processing flow is established in this article to meet the requirements to quickly handle traffic accidents. The traffic police can remotely identify accident responsibility and the insurance personnel can remotely survey an accident. Moreover, the police and insurance joint management system has been carried out in Wuhan, Central China’s Hubei Province, and Wuxi, Eastern China’s Jiangsu Province. In a word, a system is developed to obtain and analyze multisource data including precise positioning and visual information, and a solution is proposed for efficient processing of traffic accidents.

## 1. Introduction

The development of modern traffic has accelerated the development of the automobile market, but it also causes frequent traffic accidents. Not only can traffic accidents bring loss of life and property to people, but they also cause economic losses and huge time cost increases. The *Yearbook of China Transportation & Communications 2016* shows that car ownership in China reached 194 million vehicles at the end of that year and the TCI exceeds 2.0 during rush hour in some cities. There were 300,000 traffic accidents in 2016, among which the percentage of minor traffic accidents with no casualties accounted for 70% or even 80% in some cities, according to the statistics. The average time of handling such accidents is over 30 min, which is regarded as one of the major causes of traffic jams [1]. Currently, there are two typical ways to deal with traffic accidents in China. In the first way, drivers of an accident have to wait for traffic police to arrive at the scene to check and judge fault and liability. Only after that they can leave the scene. It usually takes at least two hours to complete the whole process. The other way to handle an accident is faster. Drivers take photos as evidence after an accident and then they go a designated place to deal with it. However, this can obtain evidence only after the accident but not of the whole process, which often causes disputes. More importantly, the second way cannot judge the authenticity of the accident and cannot realize remote responsibility judgement. Therefore, it is necessary to propose a scheme to effectively implement dynamic traffic monitoring and rapid accident disposal. A police and insurance joint management system is developed which based on high precision BDS/GPS positioning after combining several technologies such as the Internet, intelligent sensing, location and navigation together in a terminal and big data analysis in the remote platform. When an accident happened, it can achieve fast claims, rapid processing of traffic accidents, accurate reproduction of an accident scene and accident evidence preservation through the police and insurance joint management system.

The study motive of this article is to improve the ability of traffic control and the efficiency of handling traffic accidents. It is impossible to do the statistics of precise vehicle behaviors, remote accident responsibility identification and to identify insurance fraud based on the inadequate location accuracy. Among them, precise vehicle behaviors refer to the lane level behaviors of a vehicle such as lane changes. The judgment and statistics of precise vehicle behaviors are considered to be key to judge the fault and liability of an accident. The terminal with high precision BDS/GPS positioning can achieve precise data and realize this motive. The main contributions of this article are as follows:

1. Vehicle terminal based on high precision BDS/GPS positioning

At present, pseudo-range and Doppler measurements are usually used in GNSS vehicle navigation systems and its positioning accuracy is usually around 5 m, when without augmentation data [2,3]. This cannot meet the need of lane level behavior judgement, but it allows us to obtain an accuracy of a few meters in real-time stand-alone positioning and a handful of centimeters (i.e., four to six cm) in real-time if use of a Continuous Operating Reference Stations (CORSs) network is considered [4,5]. Therefore, the high precision positioning algorithm is integrated on the basis of the previous researches in this article, which includes Precise Point Positioning (PPP), Real-time kinematics (RTK) and inertial navigation. Multiple sensor modules fusion is also applied on the terminal. It needs to be emphasized that the terminal applies a commonly used consumer electronic device with single frequency navigation and its cost is about $20. In addition, it can access the BDS ground-based augmentation system signal, part of the CORSs network, including precise orbit data and precision clock difference data. Its positioning accuracy, which has been verified by several test experiments, can reach about 0.5 m. Thus, the terminal can provide lane level navigation and positioning service in cities.

2. Location algorithm and kernel driver

The terminal uses a high precision BDS/GPS positioning algorithm and integrated navigation algorithm. It also supports both PPP and RTK technologies, which mainly apply single frequency and real-time PPP under the requirements of IoV products. The terminal involves different insurance companies and applications which need to use big vehicle behavior data and vehicle networking. Moreover, it needs the support of high precision positioning data. However, it very difficult to master high precision satellite navigation and positioning and vehicle integrated navigation. Therefore, in this paper it is necessary to complete calculation of high precision satellite navigation positioning and vehicle integrated navigation in the Android kernel after modifying the underlying Android GPS driver. The calculation aims to realize high precision positioning when the third-party application calls the location service module. It is easier to modify GPS driver in Android rather than to realize high precision positioning on a single App. Third-party Apps can directly obtain high precision positioning results when they call the location-based service without any modification.

3. Service from police and insurance joint management system

A scheme has designed to handle minor traffic accidents rapidly is described in this article. The following data will be acquired when an accident happens: (1) the high precision trajectory data based on BDS positioning during the accident; (2) videos during a period of 10 s before and after the accident; (3) vehicle’s driving information such as driving lane, accurate rapid acceleration and rapid deceleration, sharp turns and sudden lane change; (4) the photos taken by car drivers after the accident. The four sets of data form a complete evidence chain, by which the police can analyze the scene on the remote platform and accurately simulate the accident process on a high precision map. This helps the traffic police judge fault and liability remotely and improves efficiency of handling urban minor traffic accidents. The test experiments have been carried out in Wuhan and Ningbo, which have been recognized by China’s Ministry of Public Security. Two typical actual traffic accidents among more than 30 have been selected as cases and will be explained in great detail.

## 2. Related Work

This article focuses on designing a rapid traffic accident disposal scheme based on high precision positioning, which is further developed from our previous work [6]. As the pseudo-range and Doppler measurements are usually used in the GNSS vehicle navigation system, the high-end and expensive systems can achieve real-time positioning accuracy of less than 3 m. However, the cost is too high for ordinary vehicles which only need a primary navigation module [2,3]. With the evolution of GNSSs, there are increasingly systems and enhancement algorithms for positioning technology available, and the positioning accuracy of low-cost, single-frequency GNSS receivers has been improved to a certain extent [7]. We can obtain a 4–6 cm accuracy level in real-time stand-alone positioning if a CORSs network is used [4,5,8]. This equipment uses wide area real-time PPP positioning technology. Meanwhile, it obtains enhancement data broadcast by the Chinese Beidou- based augmentation system, including precise orbit data and precision clock difference data. On this basis, the positioning accuracy has been improved to sub-meter level. Our scheme is realized through a low-cost intelligent terminal, which mainly relies on single-frequency PPP technology and supplements by RTK. The external navigation patch antenna of the terminal is installed in the front of the vehicle. Additionally, as a consumer electronic product, it applies dual-mode single-frequency BDS/GPS positioning chip and an ordinary inertial navigation sensor thus the total cost of the positioning component is less than $20.

From the obtained data of vehicle behaviors, the Usage-Based Insurance (UBI) vehicle insurance market in the United States and Europe has developed relatively maturely. They have a scheme to obtain data mainly realized by On-Board Diagnostics (OBD) models. As far as we know, Progressive Insurance is the first to obtain vehicle behaviors’ data based on OBD. It can record real time traffic data by inserting an OBD box in a car. For example, Insure The Box, an insurance company in UK that was founded in 2010, focuses on selling insurance products based on vehicle behaviors. It owns a number of OBD-based vehicle insurance patents. At present, most products in the domestic and foreign market generally use OBD interfaces to obtain vehicle data [9,10]. However, obtaining traffic data through OBD has several deficiencies as follows: (1) as the ordinary fault codes account for a small part of the total fault codes, identifying the vehicle information using the standard OBD-II interface it is very limited; (2) the installation of OBD products is very troublesome; (3) currently, OBD products obtain trouble codes mainly by Can-bus so technologies to crack the Can-bus private protocol of the original car are needed, which will result in information security risks and legal issues.

Therefore, many researchers have put forward non-OBD models to obtain vehicle behaviors, namely, to use external sensors (such as smart phones) to collect driving behavior data [9]. For example: some researchers propose to use mobile phone sensors to detect the car’s driving state, and design a way to measure driving performance indicators [11]. Others propose a framework to detect hazardous vehicle turning by using a silent Kalman filter based on the application of the GNSS measurement, and they apply it into the application of remote insurance information processing [12]. They also design a framework to deploy measurement systems based on smart phones for the application of road vehicle traffic detection and UBI vehicle insurance [13]. Moreover, some researchers analyze output data of video sensors and GPS sensors to evaluate possible risky behavior of car drivers and quality of the public transport service [14,15]. It is easy to get driving information such as location and speed from external sensors. What’s more, this can also avoid the difficulty of installing data acquisition equipment and other issues. However, these methods have deficiencies such as inadequate data accuracy, insufficient sensor fusion, single evidence chain and being non-authoritative. Therefore, this article targets the development of an intelligent terminal based on high precision BDS/GPS positioning. The terminal integrates a GPS/BDS chip, inertial sensor, network communication and other devices that it can obtain enough data for the analysis of vehicle behaviors.

As for the analysis of vehicle behaviors, most researches and analyses are conducted from the aspects of the long-term driving habits and driving risk assessment [16,17,18], but not from the aspects of claims, accident assessment and responsibility identification. This article addresses accident claims, and rapid accident responsibility identification and damage assessment.

This requires accurate judgement of lane level behaviors in order to achieve accurate vehicle behavior statistics and remote accident responsibility identification. The key point to realize the above function is to apply high precision positioning. Some researchers, such as Khaleghi et al., Yuan et al., and Tu et al., proposed to have built-in integration of multi-sensor equipment such as RTK, PPP, accelerometers, gyroscopes and magnetometers to improve the positioning accuracy and the calibration accuracy of the equipment [19,20,21]. Guo et al. suggested applying wide area precise positioning in vehicle network systems and the development of a concurrent service system with a flexible virtual extension framework to ensure reliable data exchange between the vehicle and the background, so as to realize a lane level positioning service [6]. The server can broadcast satellite- enhanced signals. Researchers utilize the signal to conduct error analysis and data processing in the space segment, communication segment and user segment of BDS/GPS satellites [22]. Thus they enhance the satellite positioning accuracy to sub-meter level for mass applications; additionally, they achieve multi-sensor fusion in the equipment and apply wide area real-time precision positioning to the vehicle network system. Therefore, they can realize high precision seamless positioning in the city after they break through various space-time barriers and solve the problem of the lack of information in location-based services. In conclusion, the accuracy of satellite positioning to sub-meter level in mass applications can be realized. In addition, it is feasible to meet the requirements of both responsibility identification and damage assessment for vehicle insurance and evidence collection for public security and traffic administration departments.

## 3. Framework

**Figure 1:**
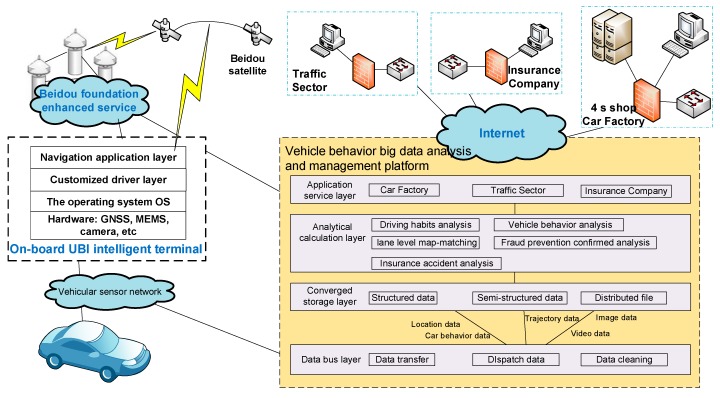
‘Terminal-Platform-Application’ trinity framework.

As shown in Figure 1, on the basis of the trinity framework, namely, ‘Terminal-Platform-Application’, high precision GPS/BDS-based technical solutions can be provided for rapid disposition of traffic accidents. A remote accident processing platform has been established. The platform includes two components: one is developed for vehicle behavior big data analysis and management in the server and the other is the data-collecting system equipped with a BDS high precision IntelliSense service [23]. The remote accident processing platform focuses on BDS wide area differential location-based services, and the analysis of high precision maps and vehicle behaviors. By being equipped with integrated multiple sensors and a sub-meter level high precision positioning module, an intelligent terminal with high precision BDS/GPS positioning can obtain high precision position and attitude information of the vehicle and upload that to the platform. At the same time, the terminal can achieve data interconnection and data sharing among insurance companies, public security and traffic administrative departments and other related institutions. That is to say, police and insurance joint management has been achieved in our framework through the combination of the user terminals and the data-analysis platform when an accident happens.

Generally speaking, when the positioning accuracy of on-board equipment cannot reach sub-meter level, it is difficult to achieve lane-level positioning. However, the users’ frequency of changing lanes or a remote judgment for the traffic accident needs the support of lane level high precision positioning. Our terminal has achieved sub-meter positioning sensing on urban roads, by integrating wide area real-time precision positioning technology and inertial navigation technology. Meanwhile, the Apps to obtain vehicle behavior big data involve a number of insurance companies and IoV applications. In order to reduce barriers for third-party developers, high precision positioning technology is equipped on the third-party Apps. High precision satellite navigation and positioning and vehicle integrated navigation have been realized in the Android kernel of the terminal. The results will be transferred to the application layer. Thus it supports the third-party Apps’ access to high precision BDS positioning data.

A sharing platform is constructed. It can integrate receiving, storage, management and release of differential data based on high precision BDS positioning, vehicle behavior data, traffic safety situation and early warning data, traffic environment and geographic information data through space-time big data analysis and collaborative computing technology. The systematic framework of the platform includes the following four parts:

1. Data bus layer

Main realization: (1) different types of data such as differential data based on BDS, vehicle behavior data, information data of insurance users and video data through different data transfer interfaces can be imported and exported. Additionally, scheduling of unified data dissemination is conducted according to their characteristics. (2) The communication/storage limitation causes data sparsity of the collected data. Thus most of the collected data cannot be analyzed and used. Therefore, it is necessary to conduct initial processing for the raw data under certain rules.

2. Converged storage layer

It can realize unified storage of heterogeneous data. Due to the different types of the collected data, the main storage components include: structured database, semi-structured database, and distributed files.

3. Analytical calculation layer

Distributed computing and dedicated processing for vehicle behavior big data have been realized. Moreover, different functions such as map matching, short time vehicle behavior analysis, long time driving habits analysis and insurance fraud protection analysis and insurance accident analysis according to the different data contents have been realized.

4. Application service layer

Data sharing has been realized among carmakers, public security and traffic administrative departments through different data transfer interfaces to provide services according to their different needs.

## 4. Key Technologies

### 4.1. Vehicle Intelligent Terminal

In this article, the intelligent terminal is based on high precision BDS/GPS positioning with sub-meter level. On the basis of this, the terminal is integrated with an inertial sensor, vehicle vision and network communication. Thus the terminal can record driving data, provide navigation and location services, realize fast claims, advanced driver assistance and so on.

**Figure 2:**
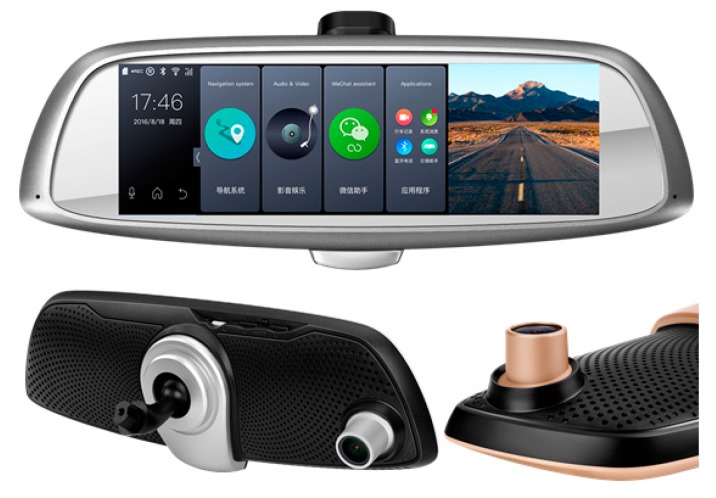
Appearance photo of our UBI terminal based on high precision BDS/GPS positioning.

As shown in Figure 2, the shape of the terminal in this article is an intelligent rear view mirror. Its standard configuration are as follows: All Winner T3 Quad-core 1.2 G ARM CortexTM-A7 high performance processing chip, assisted by a T3 Mail450 MP3GPU graphics accelerated processor, standard 4 GB~16 GB EMMC memory, 5.0 inch HD (800 × 480) highlight five point capacitive touch screen blue light anti-dazzle display, and embedded 4 G TDD/TDS/GSM/FDD/WCDMA five network communication modules, supporting vehicle HD front and back dual cameras (front camera 1080 P, 1700 wide angle, back camera VGA 576P). The terminal is an afterload device that now can be installed on mainstream vehicles.

The terminal developed in this article is an IoV product for civilian use. Crucially, it uses the single frequency navigation consumer electronic device and applies the PPP-RTK positioning method. As PPP positioning is not restricted by the distance to reference stations, it is particularly applicable to the dynamic and precise positioning field within a far and wide area and can receive modified information by radio. Therefore, PPP positioning is mainly used in this article. Moreover, the algorithms of space segment, communication segment and user segment, respectively, have been modified in order to improve the positioning accuracy to sub-meter level. The error-correcting method in space segment includes orbit dynamics model, conservative force such as Earth gravity field, non-conservative force such as solar radiation pressure and Earth radiation pressure as well as real-time forecast model. The error-correcting method in communication segment includes space environment model, spatio-temporal transformation characteristics and real-time ionospheric delay modeling. The error-correcting method in user segment includes error identification and compensation for multiple GNSS system, integration processing for observation magnitude of multiple system and real-time fast localization algorithm. The detailed algorithm implementation process will not be explained any more as it is not the main point of this article.

**Figure 3:**
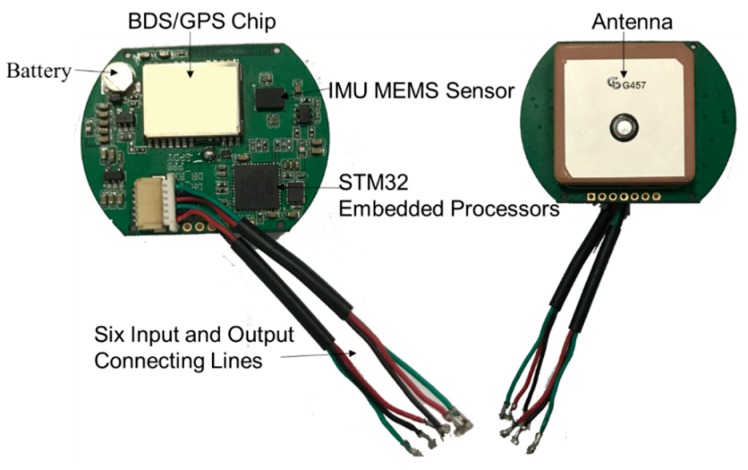
Sketch of the BDS/GPS positioning module.

The terminal accesses the BDS ground-based augmentation system signal and uses a high precision positioning module with the original observation output. It realizes calculation of high precision positioning algorithms in the Android kernel layer using a kernel positioning driver. Meanwhile, it transfers the calculation results to users through the high precision positioning interface. The Android operating system kernel driver which supports the GNSS/Inertial Navigation System (INS) integrated navigation is detailed in Section 4.2. The high precision positioning module is shown in Figure 3. The high precision positioning module consists of the following sections: battery, BDS/GPS chip, IMU MEMS sensor, STM32 embedded sensor, antenna and six input and output connecting lines. Among them, the role of BDS/GPS chip is to provide observation and ephemeris data for high precision positioning algorithm; the MEMS sensors are to provide real-time vehicle acceleration and angular velocity data.

**Figure 4:**
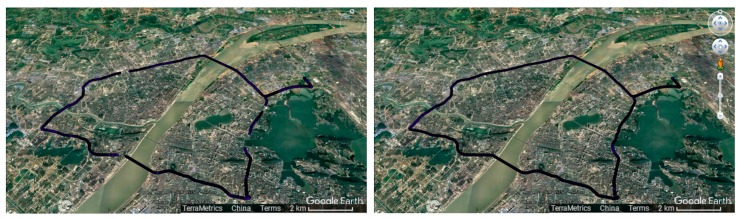
The whole vehicle trajectory on the 2nd Ring Rd; (The left one is obtained from GNSS, the right one is obtained from GNSS/INS).

The terminal’s location performance will be introduced by test experiments. A number of test experiments have conducted for a month on the 2nd Ring Rd in Wuhan. The 2nd Ring Rd is a 48-km-long expressway in the downtown area of Wuhan. 84% of the whole road is without any roadblocks and sheds and 16% of the road is a blocked section with acoustic sheds and tunnels whose length is about 300 m to 2.5 km. The whole trajectory of the 2nd Ring Rd is shown Figure 4. It is clear that the GNSS positioning cannot obtain the vehicle’s location when it drives on road with noise barriers. Thus it is necessary to use INS.

In the test experiments, the reference data is obtained from a POS 620 unit. POS 620 is a GNSS dual antenna fiber gyroscope positioning system which supports multi-GNSS (BDS, GPS and Global Navigation Satellite System (GLONASS)). It applies a six frequency GNSS receiver with high sensitivity, professional level of positioning and attitude heading, multi-channel for tracking GNSS signals and the integrated inertial measurement sensor based on high-precision quartz disturbance accelerometer and fiber gyroscope. The carrier and code pseudo-range measurements provided by the GNSS receiver and the observations of the inertial measurement sensor are combined by using a tight coupling technique. On this basis, the system can provide attitude information such as horizontal attitude and course, positioning information such as longitude, latitude, altitude and others, and inertial measurement information such as three-dimensional acceleration, angular velocity [24]. The positioning accuracy can reach centimeter level with RTK and the performance of IMU is listed in Table 1. Therefore, the POS 620 is selected as the reference in the experiments for its high accuracy.

In this article, we show three kinds of test results:

1. Accuracy evaluation in the lateral direction of the lane

**Figure 5:**
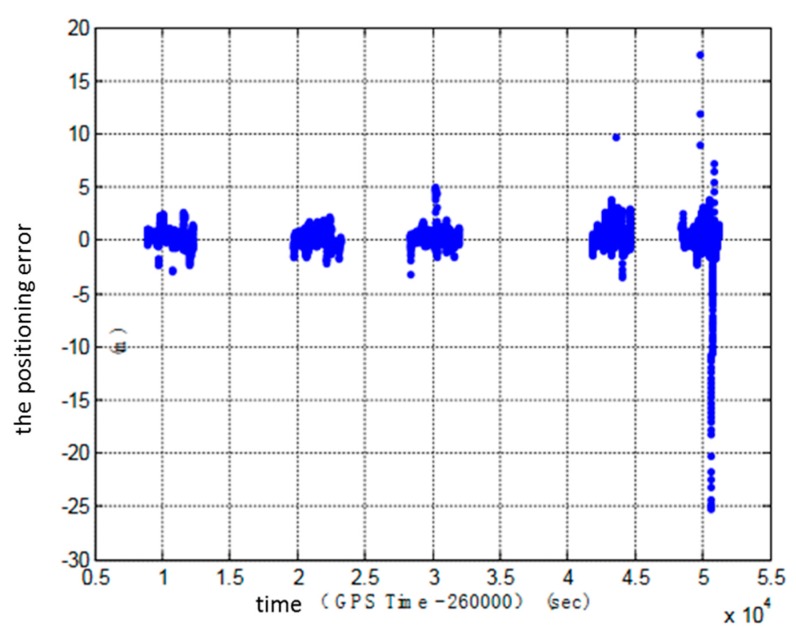
The location error (fixed solution) in the lateral direction of the lane in five tests.

The location error in the lateral direction of the lane is a key factor to judge whether a vehicle changes lanes or not. The location error of the terminal in the lateral direction of the lane in five tests is shown in Figure 5. The statistics of location error in the lateral direction of the lane are shown in Table 2.

The total number of epochs of the device in the five tests is 12,721, of which the proportion of the location error less than 0.5 m is 57.5541%; the proportion of the location error less than 1 m is 86.5277%; the proportion of the location error less than 1.5 m is 94.3217%.

2. Accuracy evaluation of lane change

The methods used are as follows: (1) display trajectories of the tested equipment and the reference equipment on the high-definition map; (2) make a comparison between the trajectories of tests and on the high definition map and judge whether the time of actively changing lane and its direction are consistent with that on the high definition map; (3) count the successful number of times of lane change and calculate the success rate of lane change. Fifteen instances of active lane changes have been recorded in each test. The statistics for successful number of times of lane change are shown in Table 3, which were obtained by comparison of the screenshots.

3. Comparison with a typical mass-market GNSS receiver

**Figure 6:**
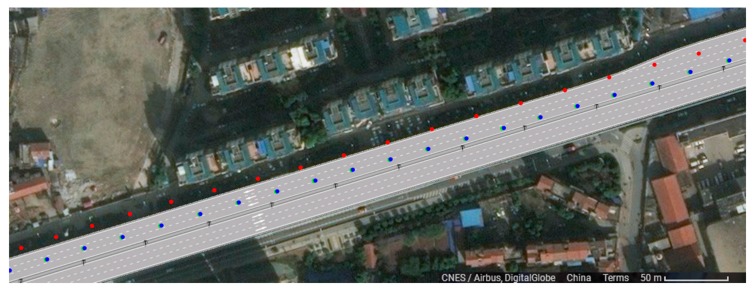
Demonstration of the vehicle trajectory on high precision map (the green one is obtained from high precision terminal; the red one is obtained from a Ublox UBX-M8030-KT; the blue one is obtained from the POS 620).

Two groups of qualitative analysis are carried out on the positioning data acquired from test experiments. The reference data obtained from POS 620 is shown as the blue trajectory in Figure 6. Our equipment is installed on the vehicle and can obtain the positioning trajectory data of track points when the vehicle is traveling. The positioning trajectory data used in the first group is provided by our equipment with high precision positioning, and indicated by the green trajectory shown in Figure 6. The data used in the second group, indicated by the red trajectory in Figure 6, is provided by a Ublox UBX-M8030-KT device, a model commonly used in the automotive application field , which applies SPP positioning chip. The contrast performance on the high precision lane level e-map is shown in Figure 6. It is obvious that the red trajectory is off the track and cannot provide lane level positioning while the green one can show which lane the vehicle drives on and whether it has any lane change behavior. The accuracy assessment of the two groups is shown in Table 4. It is clear that the positioning accuracy of the typical mass-market equipment is about 6 m. The track point drifts from side to side. We can judged which road the vehicle is driving on, but it is impossible to judge which lane it is driving in.

**Figure 7:**
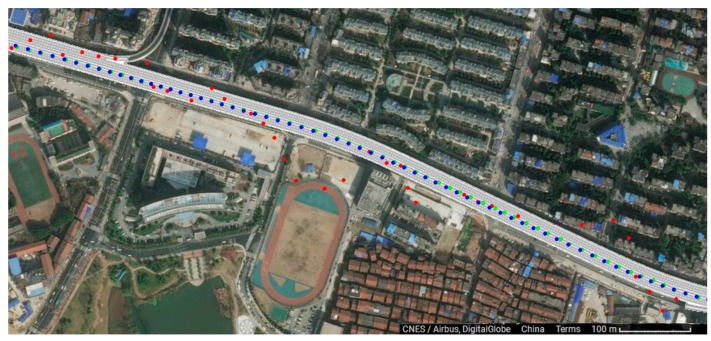
The trajectories of vehicle on roads with noise barrier (the green trajectory uses only satellite positioning, and the red trajectory uses both satellite positioning and inertial navigation, while the blue one is obtained from the POS 620 unit).

The terminal is mainly used on the urban expressway and arterial roads. Certainly, there are some sections with noise barriers and tunnels which cannot obtain satellite signals. The length of the section is less than 3 km on average in the urban traffic environment, as shown on the left of the figure. The inertial module is embedded in a high precision module and its performance has been verified as shown on the right of Figure 7. This is a 2.2-km-long section of the 2nd Ring Rd. with noise barriers. The trajectory with red dots is obtained only by satellite positioning while the trajectory with green dots is obtained by inertial navigation. The red trajectory cannot provide accurate location data while the green one can provide accurate location data when the vehicle enters the tunnel.

### 4.2. Kernel Positioning Driver

The terminal developed in this article not only involves the traffic police department and different insurance companies when it provides police and insurance joint management service. It also serves as an IoV product. Therefore, it is necessary to provide high precision positioning data for different kinds of Apps installed in the terminal, but it is very difficult for developers to master both the high precision BDS/GPS positioning and inertial navigation in developing their third-party Apps. Therefore, a driver model, namely, CarWise, is proposed in order to reduce the skill barrier for developers. The results of this scheme are as follows: (1) Third-party Android application developers can get high precision positioning results by calling the original standard GNSS ordinary positioning Application Programming Interface; (2) Existing third-party Android applications can obtain high precision positioning results without modification.

**Figure 8:**
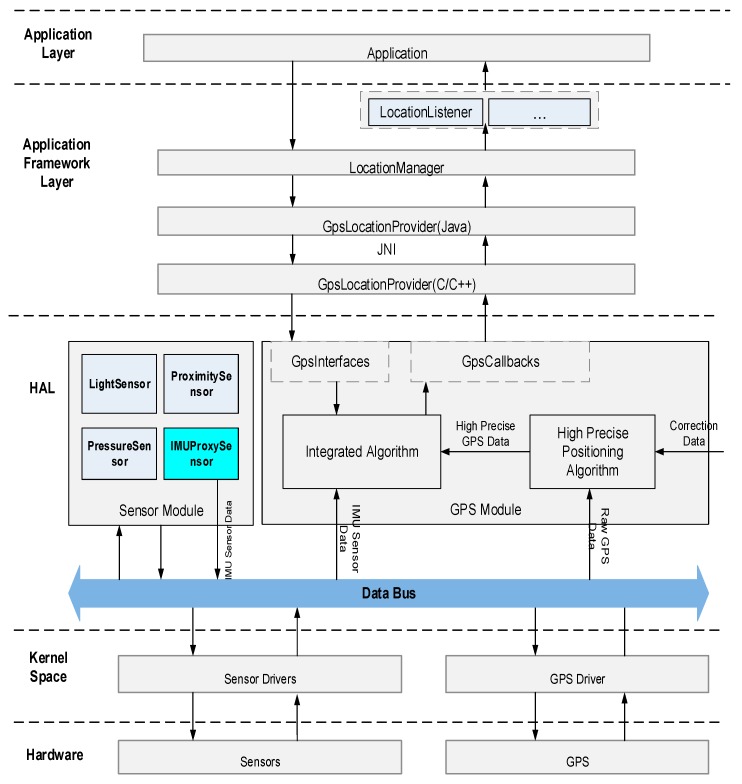
Car Wise driver model design framework.

This design scheme is to realize high precision satellite navigation and vehicle integrated navigation in the Android kernel layer of the terminal. Starting from Android operating system Hardware Abstraction Layer (HAL), upward layer by layer, it can realize the related functions of satellite high precision positioning. Based on the public architecture of the Android operating system, a high precision satellite navigation algorithm is achieved in the Android bottom layer by modifying the standard Android system GPS HAL kernel and Sensor kernel and adding a Sensor Data Agent (SDA) module to the multi-hardware HAL. Additionally, an integrated navigation positioning mode is added in the standard Android system to expand and modify a number of interface functions of related functions starting from Java Native Interface (JNI)) layer. The frame of the scheme is shown in Figure 8.

Specifically speaking, a SDA module is set for inertial navigation in the layer under the hardware HAL layer of the standard Android operating system, which guides the relevant sensor data (including 3-axis gyroscope, 3-axis accelerometer, barometer, magnetometer, optical sensors, etc.) to the Android system GPS HAL kernel and Sensor HAL kernel. To realize the relevant interfaces and data access function of the standard Android Sensor HAL kernel again can interact with the inertial sensor hardware through SDA. The standard Android GPS HAL kernel can also interact with the inertial sensor hardware via SDA. GNSS hardware data and Sensor hardware data can be directed to any HAL kernel through SDA, thus integrated navigation is realized in the kernel.

The Standard Android GPS HAL kernel is modified to support high precision positioning and an integrated navigation model. That is to say, an integrated navigation positioning implementation layer is added in the Android GPS HAL. Now the GPS HAL kernel is composed of a GNSS data access sublayer, IMU data access sublayer, high precision integrated navigation positioning sublayer and GNSS Abstract sublayer. The GNSS data access sublayer gets data from the high precision positioning module, and then coverts it into a standard GNSS data structure and puts it into a shared data area. Similarly, the IMU data access sublayer obtains the IMU sensor data through SDA, and then converts it into the standard IMU data structure and puts it into the shared data area. High precision navigation and positioning are achieved when the sublayer can copy standard GNSS data, standard IMU data and other auxiliary information data (such as magnetic compass sensor data, attitude control information, vehicle CAN bus information, etc.) from the shared data area. Then, it can do soft computation for navigation and report the calculation results to the GNSS hardware abstraction layer. GNSS abstraction sublayer has realized a package for the GpsInterface and GpsCallbacks between the sublayer and application framework layer of integrated navigation positioning, where GpsInterface is the function interface that sublayer provides for the upper application of integrated navigation positioning, GpsCallbacks is the callback function interface for downward registration of the upper application. GNSS abstraction sublayer has realized the separation of interface and implementation, making the whole architecture has better scalability.

**Figure 9:**
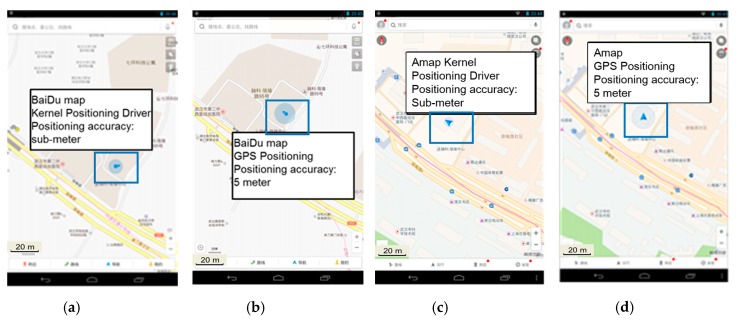
Effect contrast between Baidu Map and AutoNavi with high precision kernel driver and ordinary positioning. (**a**) Baidu Map-high precision kernel driver; (**b**) Baidu Map-GPS positioning; (**c**) AutoNavi-high precision kernel driver; (**d**) AutoNavi Map-GPS positioning.

The implementation of this method needs to rewrite GPS driver underlying Android, which is much more difficult than simply achieving high precision positioning for a certain App. An App with location-based service is selected to verify the positioning performance, and its positioning effect is shown in Figure 9. The left image shows the positioning effect of this App installed on an ordinary mobile phone, and the right image shows that of the App installed on our terminal. As shown in the figure, the size of the circle that represents the positioning precision is quite smaller, and the location is more precise.

### 4.3. Lane Level Map and Matching Technology

In the process of vehicle behavior judgment and rapid accident processing, it is necessary for the system to screen accident evidence. Thus, it is of great importance to match the high precision track of the accident with a lane level map. The match helps to identify whether the vehicle has some behaviors such as lane changes and reproduce the accident scene accurately. Afterwards a whole picture of the accident is obtained to accurately assess the accident responsibility.

In order to develop a steady model of map matching of high precision tracks, there are two main problems to be solved in this process: (1) matching and correction of coordinate coherence for map road network data and BDS/GPS track data; (2) lane matching of BDS/GPS point and discovery of lane changing.

*Definition*: The high precision road network contains lane collection L = {l_1_, l_2_, l_3_,…, l_m_}; the track data of a vehicle to be analyzed tri = {p_1_, p_2_, p_3_,…, p_n_}, is composed by a series of coordinates p_i_ (x_i_, y_i_, t_i_) with timestamp, where x_i_ and y_i_ represent the space coordinates of vehicle at time t_i_. Some high precision track data also includes semantic information of speed, direction, etc.; after matching and correction, the GPS track data Tri’ = {p_1_, p_2_, p_3_,…, p_n_}.

For the two problems mentioned above, we design a technical route. The inputs are road map data and BDS/GPS positioning track data of the vehicle; the output is the lane information corresponding to each BDS/GPS point as well as the abnormal characteristics reflected by a part of vehicle track data (e.g., “lane changing”). The lane level map matching process consists of three parts:

(1) Data preprocessing: mainly refers to conducting structured process for the data object to meet the latter analysis and calculation.
(2) Matching and correction of data consistency: the road data of based drawing and vehicle track data may be not consistent, so it is necessary to use the method of partial affine transformation to deal with the inconsistency.
(3) Lane matching and lane changing discovery: BDS/GPS track point has been matched with lane information, and the abnormal information such as lane changing has been detected. Therefore, the following algorithm is proposed:

GPS point lane matching algorithm:

1. Input: vehicle BDS/GPS positioning track data tri = {P_1_, P_2_, P_3_,..., P_n_}. And map data of road L = {l_1_, l_2_, l_3_,..., L_m_}.
2. Output: the corresponding lane information of each BDS/GPS point as well as the anomalous features reflected by a part of vehicle tracks (e.g., “lane changing” behavior).

Detailed steps:

1. Take P_i−1_ before the point P_i_, connect the two points to get segment p_i_p_i−1_;
2. Making two rays r_1_ and r_r_ on both sides of P_i_ point; the rays are perpendicular to p_i_p_i−1_; the ray length is radio; (radio value is generally 1.5 times the maximum lane width);
3. Use r_1_ to detect the lane line that intersected with r_1_ on the left side; If there are two or more different lane lines, take the shortest distance between the lane line and p_i_ point as the smallest lane line;
4. Use the same method of Step 3 to calculate the lane line that intersected with r_r_ on the right side;
5. After obtaining the lane lines of p_i_ points on both sides, extract relevant properties of two lane lines respectively. If the two lines share common lane number, then the lane number is lane that p_i_ point located; Otherwise, it fails to calculate the p_i_ point lane information; analyze and calculate the next point p_i+1_ after identifying the result;
6. Repeat steps 1–5; analyze and calculate the results after traversing each GPS point. If the lane line information of adjacent two points such as p_j_ and p_j+1_ is different, extract the information and identify as lane changing position.

## 5. Actual Accident Cases

With the development of science and technology, there are a lot of schemes for rapid processing of traffic accidents on the market. Nevertheless, almost all of them can only obtain evidence after an accident. For example, the car owners have to get off the road and take photos to get evidence after an accident. The whole process of the accident can’t be obtained. Hence, it is impossible for authorities and insurers to judge the authenticity of an accident. What’s more, when car owners are involved in a disputed accident, it is still unavoidable to wait for the police and insurance personnel to arrive at the scene.

The rapid processing scheme proposed in this article is based on high precision BDS/GPS positioning. It can obtain the whole process of an accident as evidence to judge the authenticity of the accident. It can also realize remote view of related accidents’ cars in their accurate driving situation. The scheme has been verified on a large scale in Wuhan, Central China’s Hubei Province, Ningbo, Eastern China’s Zhejiang Province and other cities. The scheme is realized through a police and insurance joint management system which has put into use since April 2017. More than 30,000 terminals have been installed and put into use. A total of over 30 traffic accidents have been recorded on the remote platform. Two typical cases have been selected in order to describe how the system works to meet the requirements of traffic police and insurance companies to deal with an accident.

1. Data upload

When an accident occurs, firstly, the system obtains high precision trajectory data based on BDS positioning during the accident; secondly, the system automatically uploads videos taken during a period of 10 s before and after the accident happened; thirdly, the system conducts an intelligent driving behavior analysis of the owners, including driving information such as driving lane records, accurate rapid acceleration and rapid deceleration data, sharp turns, sudden lane changes; fourth, the car owner gets out, takes photos and uploads the data. The four sets of key data constitute a complete chain of evidence. This truly and accurately records the scene.

2. Remote view of the trajectory

**Figure 10:**
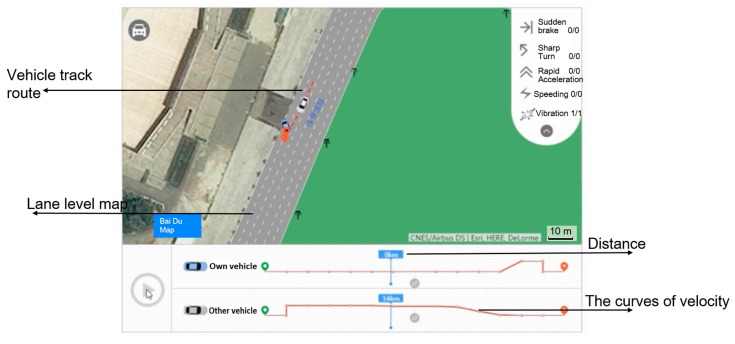
Reconstruction of the accident process.

On the remote platform, the traffic police conduct a preliminary analysis of the driving behaviors and the accident situation by combining the high precision lane level map and the accident information. The trajectory data can be viewed on the remote platform. It is a traffic accident caused by a lane change, as shown in Figure 10.

The base map is formed by overlaying the satellite map and the lane level e-map with decimeter accuracy. Then the trajectory data is matched with the base map. It is clear that the location of two cars overlays the crash point exactly. The red trajectory shows clearly the driving route of the white car. Therefore, the white car takes full responsibility for the accident as the red trajectory shows that the crash is caused by a lane change of the white car.

**Figure 11:**
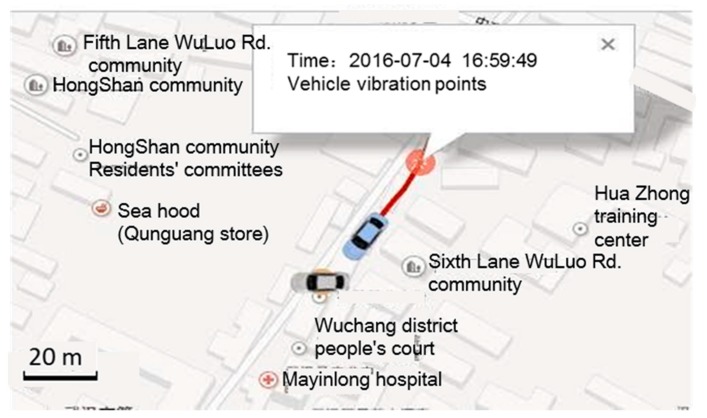
The situation of two ordinary GPS-based cars after an accident.

Currently, the accuracy of the ordinary positioning equipment on the market is not sufficient. The relevant cars are far from each other on the platform when an accident happened, and they even may not appear in the same region. The effect can be seen in Figure 11 after matching the trajectory data obtained from an ordinary mobile phone with a Baidu map as shown in Figure 11. It is obvious that the crash points are not near to the cars due to the inaccurate positioning accuracy. It is impossible for traffic police to judge the authenticity and fault and liability of the accident if they didn’t arrive at the scene.

3. Evidence chain

**Figure 12:**
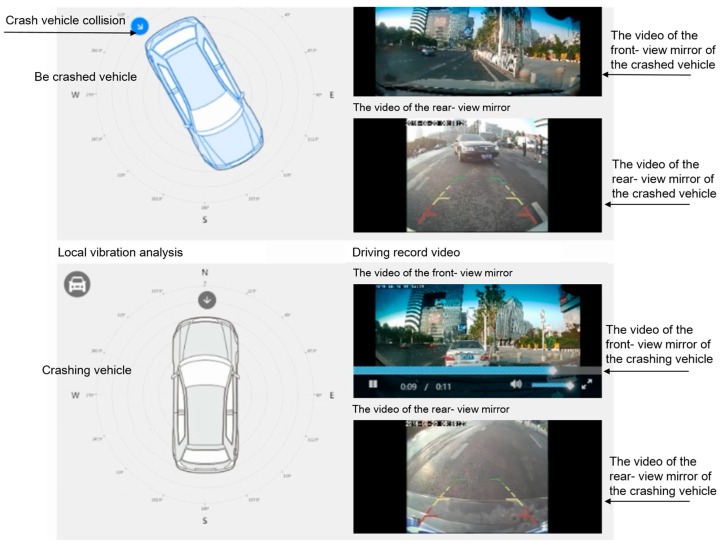
Views from the driving record video.

Certainly, the trajectory data is key evidence for the accident. On the remote accident handling platform, the traffic police can obtain more evidence: such as the vehicle vibration location when the accident happened, velocity, collision angle and time, videos during a period of 10 s before and after the accident and so on, as shown in Figure 12.

**Figure 13:**
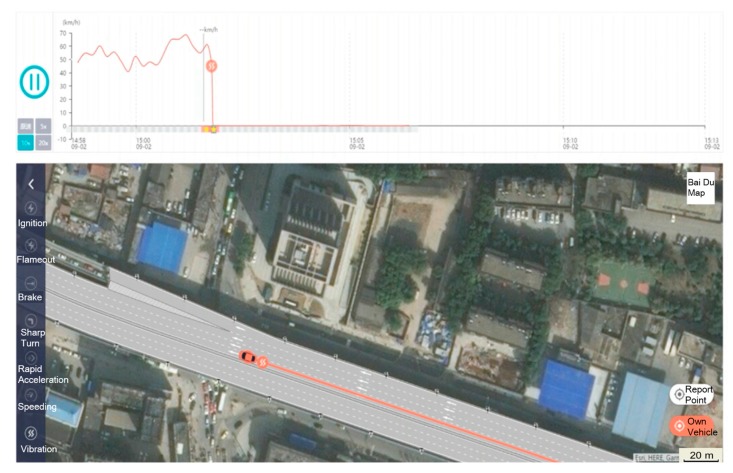
Accident simulation video.

As shown in Figure 13, this is a car rear-end collision that occurred on the 2nd Ring Rd in Wuhan. The collision point, braking point, the speedometer, and the simulated video, etc., of the accident are shown in the figure. The traffic police can know the whole process of the accident, determine the authenticity of the accident and judge the fault and liability accurately according to the relevant evidence available on the remote platform.

4. Realize rapid responsibility identification

Finally, the responsibility can be identified and the authenticity of the accident can be judged according to the data analysis of the whole accident and communication analysis between the traffic police and the relevant drivers of an accident. A location-based service application is also applied during the whole process. With this result, the owners can supplement the relevant evidence by mobile App. It also ensures that the accident scene can be restored; evidence can be collected and law can be applied.

## 6. Conclusions

This article has developed a vehicle terminal with high precision BDS/GPS positioning and a police and insurance joint management system with big data analysis and management to provide services for traffic police and insurance companies. It represents a breakthrough in the application of wide area real-time high precision positioning in vehicle systems. A solution scheme is also proposed to realize the fusion of multi-source data including the space-time data of BDS/GPS and multiple sensors and the visual data of the accident scene. The scheme has satisfied its goal to achieve remote responsibility identification and damage assessment, accurate reproduction of the scene, preservation of complete evidence, prevention of insurance fraud, police and insurance joint management, rapid claims to insurance companies and quick evacuation of the scene. The existing accident treatment way can obtain the evidence only after an accident, which cannot obtain the complete evidence and judge the accident’s authenticity. However, the intelligent terminal developed in this article can achieve all that. This multi-functional intelligent terminal, with integrated functions of driving records, navigation location-based services, fast claims, advanced driving assistance system and vehicle information services, has been deployed in Wuhan, Wuxi, Ningbo and other cities in China on a large scale. It can promote commercial vehicle insurance reform and technical innovation in China.

Although the application of the intelligent terminal in handling minor traffic accidents is emphasized in this article, the terminal can also be applied in other fields, such as the insurance industry. The terminal can accurately obtain the vehicle’s behavior information, including acceleration, deceleration, sharp turns, and sudden lane change and so on and can conduct statistical analyses of these data. UBI insurance mode is a differentiated vehicle insurance based on users’ different driving behaviors, and the vehicles’ behavior information is necessary in this mode. It is also a development trend of insurance mode. At the same time, the vehicle behavior data can help design new types of insurance. Moreover, it can be applied to construct an actuarial model and calculate price premium rates using vehicle lane level behavior data, which will be our goal for further study.

## Figures and Tables

**Table 1:** Characteristics of the IMU.

Characteristics		Tactical
Gyroscope	bias (1σ)	0.1°/h
	white noise (ARW)	0.03${}^{\circ}/\sqrt{h}$
	scale factor (1σ)	200 ppm
Accelerometer	bias (1σ)	25 mGal
	white noise (VRW)	0.05 $\frac{m}{s}/\sqrt{h}$
	scale factor (1σ)	200 ppm

**Table 2:** Statistics of location error in the lateral direction of the lane.

Tests	The Number of Epochs of the Location Error Less than 0.5 m	The Proportion of the Location Error Less than 0.5 m	The Number of Epochs of the Location Error Less than 1 m	The Proportion of the Location Error Less than 1 m	The Number of Epochs of the Location Error Less than 1 m	The Proportion of the Location Error Less than 1.5 m
The 1st lap	1220	45.74428%	2435	91.30109%	1811	97.41282%
The 2nd lap	1602	60.79696%	2289	86.86907%	2069	95.59772%
The 3rd lap	2108	69.59393%	2850	94.09046%	2996	98.91053%
The 4th lap	1186	51.6101%	1773	77.15405%	2519	90.03481%
The 5th lap	1202	57.62224%	1655	79.33845%	2598	86.81687%

**Table 3:** Statistics table for successful number of times of lane change.

	Number of Successful Lane Changes
The 1st test	15
The 2nd test	14
The 3rd test	12
The 4th test	13
The 5th test	12
Total number of successful lane changes	66
Lane change success rate of	88%

**Table 4:** Accuracy assessment of the two methods.

Total Number of Epochs: 4064	Lateral Deviation RMS (m)	Lateral Deviation MEAN (m)	Max Lateral Deviation	Longitudinal Deviation RMS (m)	Longitudinal Deviation MEAN (m)	Max Longitudinal Deviation
UBX-M8030-KT	5.022	1.288	67.658	18.053	3.794	170.931
terminal	0.380	0.294	1.307	0.438	0.353	1.305
